# Assessing the potential of NS2B/NS3 protease inhibitors biomarker in curbing dengue virus infections: *In silico* vs. *In vitro* approach

**DOI:** 10.3389/fcimb.2023.1061937

**Published:** 2023-02-14

**Authors:** Harun Norshidah, Chiuan Herng Leow, Kamarulzaman Ezatul Ezleen, Habibah A. Wahab, Ramachandran Vignesh, Azhar Rasul, Ngit Shin Lai

**Affiliations:** ^1^ Institute for Research in Molecular Medicine (INFORMM), Universiti Sains Malaysia, Penang, Malaysia; ^2^ Universiti Kuala Lumpur-Royal College of Medicine Perak, Ipoh, Perak, Malaysia; ^3^ School of Pharmaceutical Sciences, Universiti Sains Malaysia, Penang, Malaysia; ^4^ Department of Zoology, Faculty of Life Sciences, Government College University, Faisalabad, Pakistan

**Keywords:** dengue virus, NS2B/NS3pro, antiviral, drug discovery, diagnostics

## Abstract

An increase in the occurrence of viral infectious diseases is a global concern for human health. According to a WHO report, dengue virus (DENV) is one of the most common viral diseases affecting approximately 400 million people annually, with worsening symptoms in nearly 1% of cases. Both academic and industrial researchers have conducted numerous studies on viral epidemiology, virus structure and function, source and route of infection, treatment targets, vaccines, and drugs. The development of CYD-TDV or Dengvaxia^®^ vaccine has been a major milestone in dengue treatment. However, evidence has shown that vaccines have some drawbacks and limitations. Therefore, researchers are developing dengue antivirals to curb infections. DENV NS2B/NS3 protease is a DENV enzyme essential for replication and virus assembly, making it an interesting antiviral target. For faster hit and lead recognition of DENV targets, methods to screen large number of molecules at lower costs are essential. Similarly, an integrated and multidisciplinary approach involving *in silico* screening and confirmation of biological activity is required. In this review, we discuss recent strategies for searching for novel DENV NS2B/NS3 protease inhibitors from the *in silico* and *in vitro* perspectives, either by applying one of the approaches or by integrating both. Therefore, we hope that our review will encourage researchers to integrate the best strategies and encourage further developments in this area.

## 1 Introduction

Dengue virus (DENV), an RNA virus belonging to the family *Flaviviridae* and genus Flavivirus, is a fatal pathogenic arthropod-borne virus (arboviruses). It is predominantly transmitted by *Aedes aegypti* and, to a lesser extent, *Aedes albopictus*. The disease is widespread in more than 110 countries, infects approximately 400 million people, and results in approximately 20,000 deaths annually (Liang Gao and Gould, 2015; World Health Organization, 2020). Over the past few years, the occurrence of dengue fever (DF), dengue hemorrhagic fever (DHF), and dengue shock syndrome (DSS) has significantly increased in major tropical regions, with alarming frequency, magnitude, and bearing dire consequences (Tiga-Loza et al., 2021). DENV has four antigenically distinct serotypes (DENV1–DENV4) with 65–70% identical genome sequences. Each DENV serotype comprises four–seven genotypes that differ by 10% at the amino acid level across the envelope protein. The four serotypes differ not only in sequence similarity but also in infection dynamics. For example, DENV-1 is the most common serotype, followed by DENV-2, which is more frequently associated with severe infections. However, the mechanisms underlying dengue infections, as well as the entire set of distinctions across serotypes, remain unknown. However, a few recent studies have investigated the differences between the serotypes (Delli Ponti and Mutwil, 2021; Katzelnick et al., 2021; Stica et al., 2022). In light of the above, we hope that the source, breadth, and impact of antigenic heterogeneity can be better understood, which will aid in the exploration of effective dengue inhibitors or vaccines.

One of the major milestones in combating dengue infection was the first licensed vaccine, CYD-TDV or Dengvaxia^®^. Nevertheless, owing to some drawbacks and limitations in the ongoing trials, it was found that the vaccine increased the risk of developing a severe form of dengue infection in some receivers (Redoni et al., 2020; Tully and Griffiths, 2021). This has led researchers to accentuate the development of potent inhibitors that can curb infection. Therefore, it is crucial to explore drugs directed at viral targets or critical host mechanisms that can be used as prophylaxis or treatment for the disease. Drug efficacy in the effective amelioration of the disease or the reduction of disease severity and fatalities is needed to lower the burden of dengue (Low Gatsinga et al., 2018).

Pharmacological interventions for DENV replication can be targeted for antiviral treatments. Over the years, DENV enzymes, such as NS2B/3 protease (Yusof et al., 2000; Leung et al., 2001; Li et al., 2005; Erbel et al., 2006), NS3 helicase/NTPase/RTPase (Egloff Benarroch et al., 2002; Wang et al., 2009; Basavannacharya and Vasudevan, 2014), NS5 methyltransferase (Lim et al., 2008; Lim et al., 2011; Barral et al., 2013; Lim et al., 2013), and NS5 polymerase (Nomaguchi et al., 2003; Selisko et al., 2006; Niyomrattanakit et al., 2010; Niyomrattanakit et al., 2015) have been studied comprehensively for pharmacological intervention. Among these enzymes, NS2B/3 protease, which plays multiple roles in the viral life cycle, is an attractive target for dengue antiviral drug discovery. One of the methods for faster hit and lead recognition for DENV targets is to screen a large number of chemical molecules using high-throughput screening at lower costs. An integrated and multidisciplinary approach that integrates biochemical approach and virtual simulations are frequently used in drug discovery.

**Figure 1:**
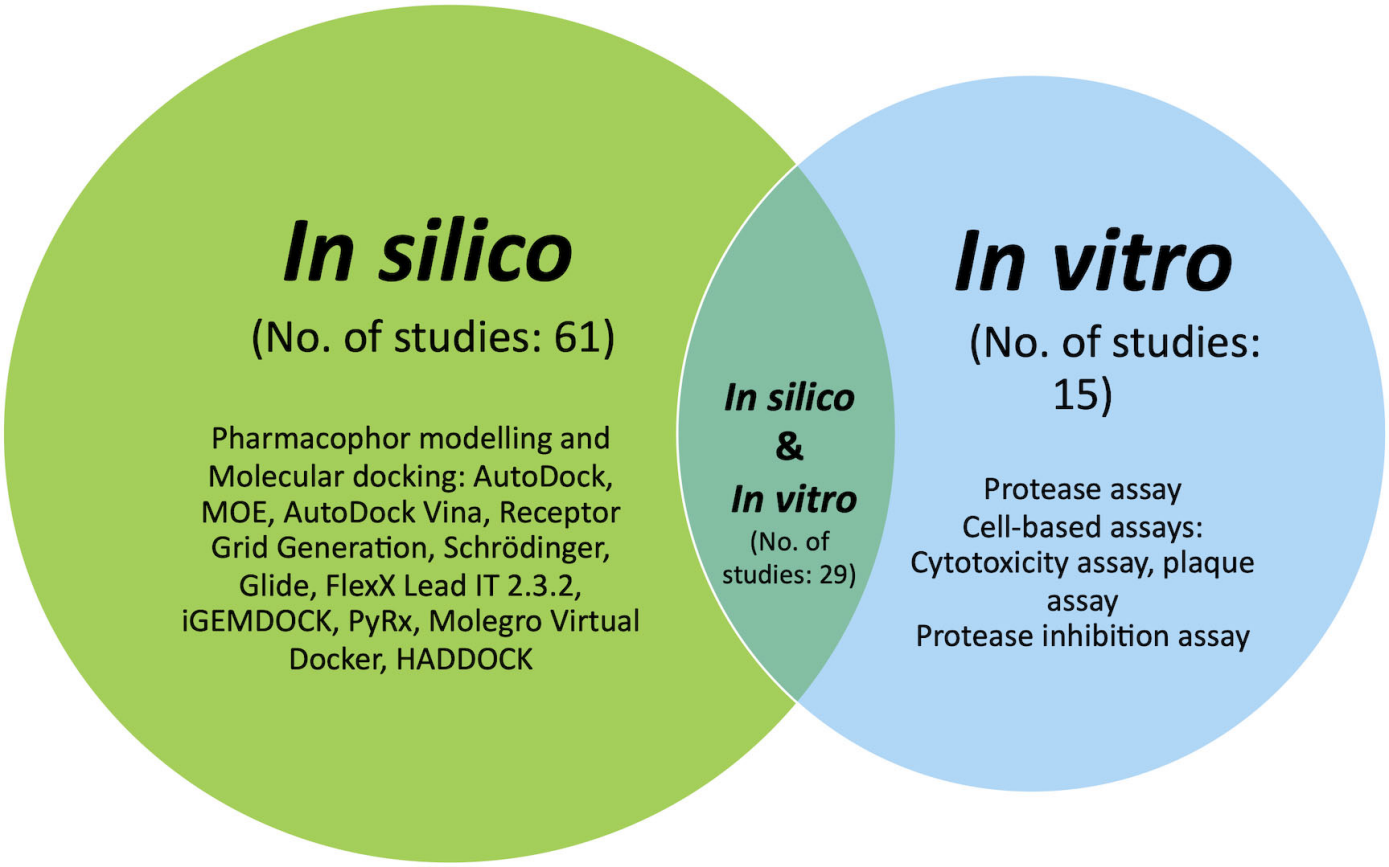
Venn diagram illustrating the distribution of the *in silico*, *in vitro* and approach integrating both strategies.

This study examined 105 studies published in the Scopus citation database, MEDLINE, PubMed, and Google Scholar from 2015 to 2022. The indexed articles focused on discovering potential DENV NS2B/NS3 protease inhibitors using *in silico* and *in vitro* approaches, either by integrating both or applying one of them. Hence, search strings tailored to each database were devised for the dengue NS2B/NS3. Mendeley (Elsevier, London, England) was used to compile references for the identified articles, and duplicates were removed. All identified abstracts were examined and selected based on preset criteria. A systematic review of this paper began by tabulating significant potential inhibitors in *silico* and *in vitro* studies, followed by a Venn diagram illustrating the strategy distribution (**Figure 1**). This review seeks to explore a better understanding of NS2B/NS3 proteases and their therapeutic inhibitory potential and thus enlighten researchers on integrating the best strategies in this area.

## 2 DENV polyproteins

**Figure 2:**
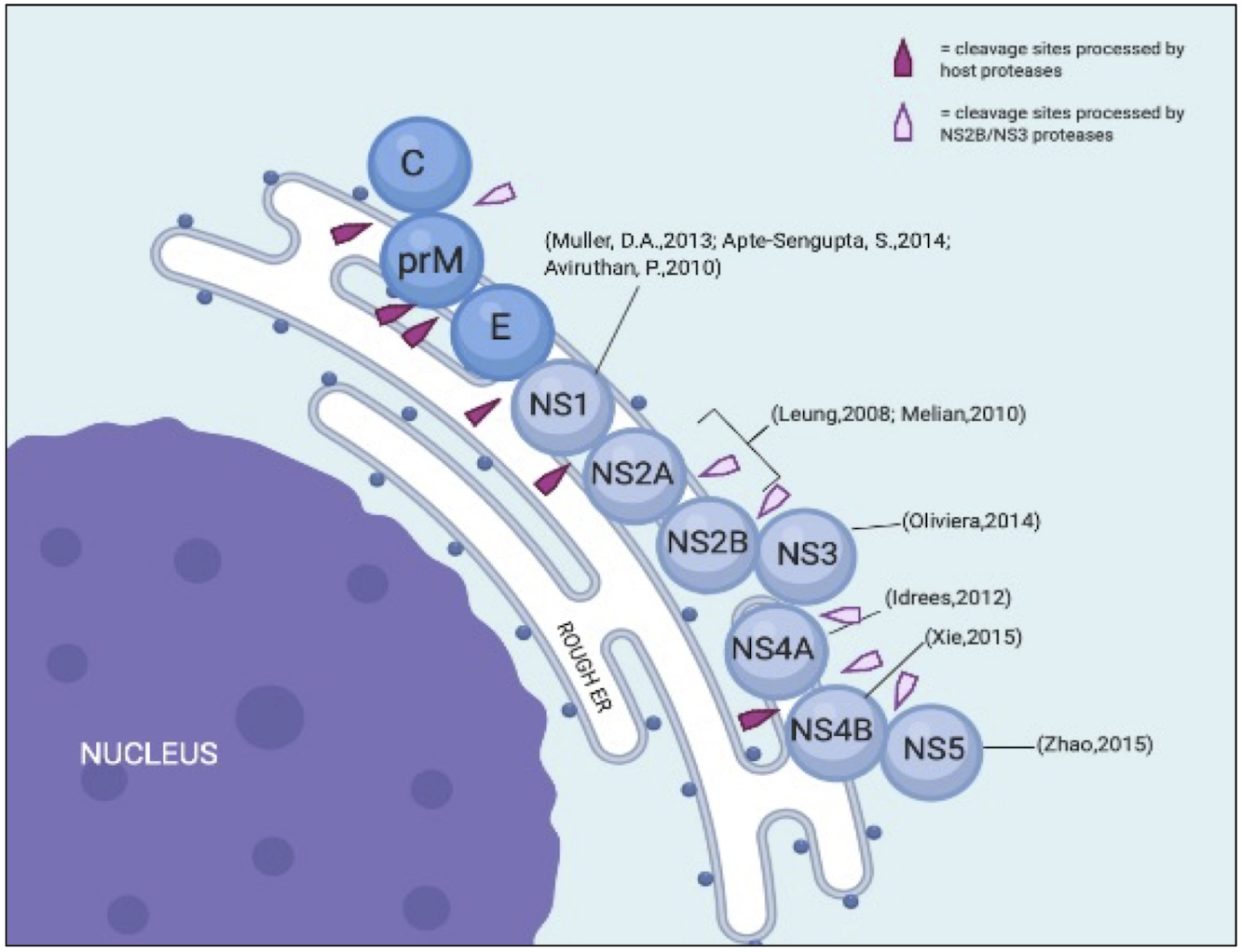
Illustration of DENV structural and NS proteins on the ER with its cleavage sites. Modified from Uno & Ross, 2018.

Morphologically, dengue viruses are approximately 50 nm in diameter with an open reading frame (ORF) of over 10,000 bases (Hahn et al., 1990). Upon infection, the positive-sense single-stranded RNA genome is replicated and translated in the endoplasmic reticulum (ER), where host ribosomes translate RNA into polyproteins. These nascent proteins are further broken down by host and viral proteases into structural and nonstructural (NS) proteins (**Figure 2**).

The ORF is flanked by two untranslated regions (UTRs) that contain structural and functional elements essential for viral translation and replication. The UTRs are translated into polyproteins that are processed co- and post-translationally by the host and DENV proteases to produce ten mature viral proteins. From the *N*-terminal region, three structural proteins are encoded in the N-terminal region: capsid protein (C, 11kDa), membrane protein (M, ~8kDa), and envelope proteins (E, 53 kDa) (Hosseini et al., 2018). NS proteins are essential for viral replication and are retained in all DENV serotypes (Ahmad and Poh, 2019). Hence, these proteins are important components of the DENV genome replication machinery. **Table 1** briefly describes each protein and its relevance to viral pathogenicity.

**Table 1:** Brief description of dengue NS proteins.

Non-structural Protein	Description	References
NS1	A 46kDa glycoprotein. At the start of the infection process, interacts with NS4A and NS4B transmembrane proteins.	(De Clercq, 2009; de Sousa Wu et al., 2015; Dhar Dwivedi et al., 2020)
NS2A	A hydrophobic transmembrane protein with 22-kDa and 218 amino acids. The N-terminal contains 68 amino acids in the lumen of the ER whereas the C-terminal located at the cytoplasm contains 10 amino acids.	(Egloff Benarroch et al., 2002; Dwivedi et al., 2016; Dražić et al., 2020)
NS2B	A co-factor to NS3 protease. A hydrophobic protein with 15-kDa (130 amino acids).	
NS3	~69kDa multifunctional enzyme acts protease RNA triphosphatase and helicase.	(Oliveira Silva et al., 2014)
NS4A	~16kDa; highly hydrophilic on the end of its C-terminus. Suitable as a signal for translocating NS4B to ER lumen.	(Idrees and Ashfaq, 2012)
NS4B	Consists of 248 amino acids. Small integral membrane protein with high hydrophobicity.	(Xie Zou et al., 2015)
NS5	104kDa, largest NS protein. Bi-functional enzyme; N-terminal is the domain of methyltransferase and C-terminal is the polymerase RNA dependent on the RNA.	(Zhao et al., 2015)

### 2.1 DENV NS2B/NS3 protease as drug target

**Figure 3:**
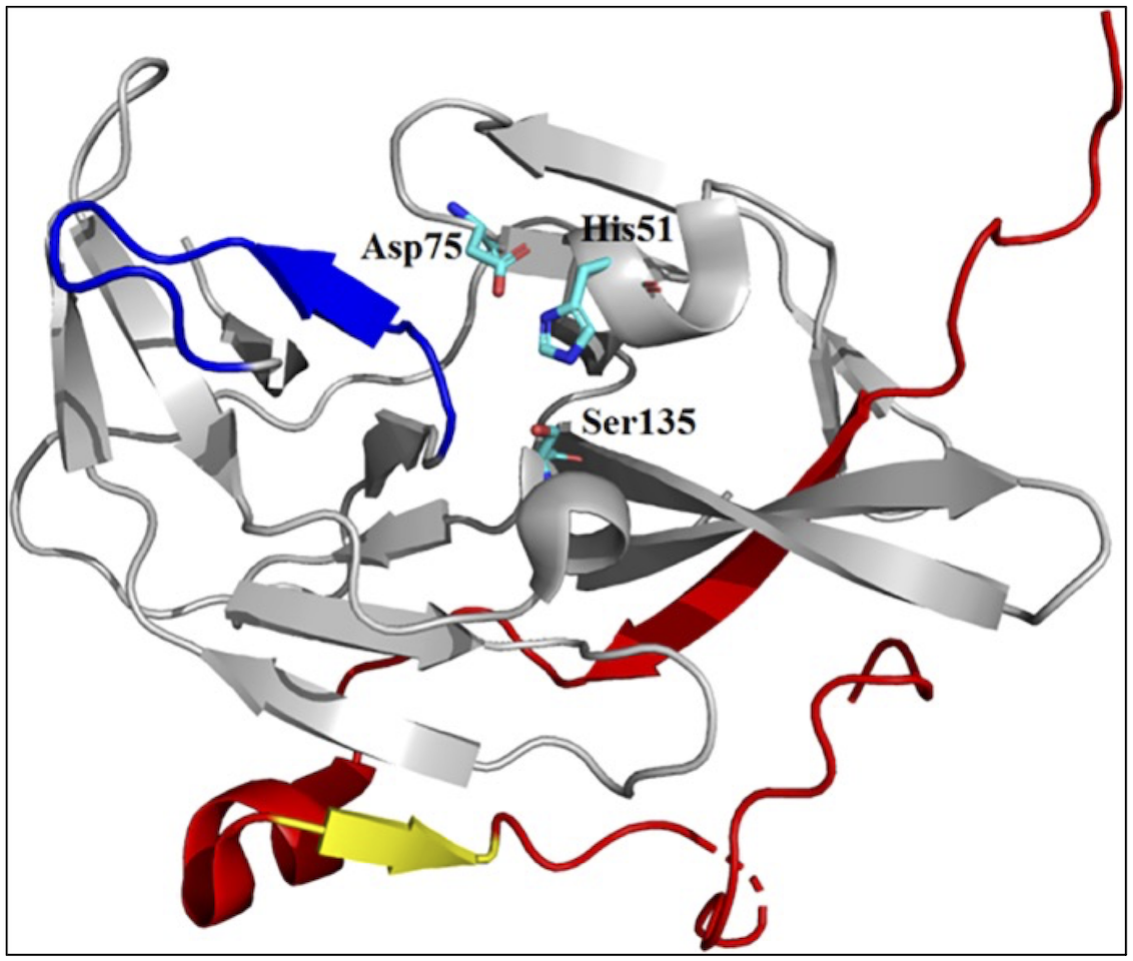
X-ray crystal structure of catalytically active conformation of DENV NS2B/NS3 pro (PDB code: 2FOM). Grey ribbon: NS3 structure; red ribbon: NS2B cofactor; yellow ribbon: S1-ß-hairpin; and blue ribbon: ST-loop. Figure adapted from Erbel et al. (2006).

NS3 is a large multifunctional protein with serine protease (with NS2B as a cofactor), 5′-RNA triphosphatase (RTPase), nucleoside triphosphatase (NTPase), and helicase activity (Wengler and Wengler, 1991; Warrener et al., 1993; Li et al., 1999). The N-terminal 170 amino acids of NS3 have protease activity and a hydrophobic core of approximately 40 amino acids within NS2B that provides an essential cofactor function (Hahn et al., 1990; Chambers et al., 1991; Falgout et al., 1991). NS3 protease (NS3 pro) is a trypsin-like serine protease with a classic serine protease catalytic triad consisting of His51, Asp75, and Ser135 residues (Bazan and Fletterick, 1989). All four DENV serotypes have approximately 65–74% amino acid sequence homology and a common substrate preference (Li et al., 2005). The C-terminal β-hairpin of NS2B in its catalytically active form wraps around the active site of NS3 (**Figure 3**) (Erbel et al., 2006). Consistent with the important structural role of the C-terminal β-hairpin of NS2B, structural comparisons indicated that the amino acids within the N-terminal portion displayed similar conformations in all structures, regardless of the presence or absence of inhibitors.

### 2.2 Prospective developments

Considering the global threat of DENV and the urgent need for effective drugs, several efforts have been made to identify potential protease inhibitors. The development of NS2B/NS3pro inhibitors began with the structure-activity relationship of NS2B-NS3pro, inferred from the well-established cleavage sites of the DENV polyprotein by NS2B-NS3pro. This led to the discovery of two tetrapeptides, Bz-Nle-Lys-Arg-Arg and Bz-Nle-Lys-Thr-Arg, that have been shown to have high affinities for NS2B-NS3pro (Ki¼12.42 and 33.9 mM, respectively) (Yin et al., 2006; Othman et al., 2007; da Silva-Júnior and de Araújo-Júnior, 2019). Subsequently, efforts have been made to design peptidomimetics that have the ability to mimic the natural substrate (Gibbs et al., 2018; da Silva-Júnior and de Araújo-Júnior, 2019; Dražić et al., 2020).

The latter group of inhibitors has long been recognized as an invaluable component of medicine, and many targeted therapies have focused on these small-molecule drugs. These low molecular weight (less than 900 Da) organic compounds help to control biological targets, such as enzymes, channels, or receptors, to alter the disease cycle (Phanthanawiboon et al., 2014; Lenci and Trabocchi, 2019). At present, 90 percent of the therapeutics in the pharmaceutical market are small-molecule drugs. These include ten clinically available human immunodeficiency virus 1 (HIV-1) protease inhibitors and hepatitis C virus (HCV) protease inhibitors (De Clercq, 2009; Manns and Von Hahn, 2013). These facts also suggest that protease inhibitors of the dengue virus could be clinically effective. In the last decade, the development of small molecule NS2B/NS3pro inhibitors has involved high-throughput screening (HTS) of the natural product (Kiat et al., 2006; de Sousa Wu et al., 2015), and synthesis of rational drug design (Liu et al., 2014; Viswanathan et al., 2014; Raut et al., 2015a) with virtual screening using computer-aided drug design (CADD) being in-process (Cabarcas-Montalvo et al., 2016).

This review highlights the recent development of DENV inhibitor successors, mainly small molecules. Owing to advances in bioinformatics in drug discovery, non-peptide antiviral activity evaluation has been explored *in vitro*, as well as *in silico* and in HTS (Kanakaveti et al., 2020). Weighing the benefits of both approaches provides greater knowledge and an understanding of the anti-DENV drug development pipeline. Summarizing our findings on the methods used in developing NS2B/NS3pro inhibitors, this study highlights methods that are relevant to this co-protein only. Methods were classified into three cohorts: studies focusing on *in silico* methods, *in vitro* methods, and both. By subdividing these approaches, we hope this analysis will promote further progress in discovering potent inhibitors of fatal arbovirus infections.

## 3 *In silico* approach

Various computational tools have been used to identify small target molecules for dengue drug discovery. Structure-based drug design (SBDD) methods, namely molecular dynamics, fragment-based drug design, pharmacophore modelling, and most importantly, molecular docking, have provided information about many molecules, including DENV protein targets, such as NS2B/NS3pro. Among the above mentioned methods, molecular docking is the most popular for searching for potential NS2B/NS3pro inhibitors. The aim of molecular docking is to determine the best ligand-binding positions in the NS2B/NS3pro binding pocket and estimate the affinity of the ligand for the protein (Jakhar et al., 2019). To date, 13 crystal structures of DENV NS2B/NS3pro with different PDB codes have been solved for all dengue serotypes (**Table 2**).

**Table 2:** PDB codes of NS2B/NS3pro crystal structure for all DENV serotypes.

Dengue serotypes	NS2B/NS3pro PDB code	References
DENV1	3LKW, 3L6P	(Chandramouli et al., 2010)
DENV2	2FOM, 4M9T, 4M9K, 4M9M, 4M9I, 4M9F	(Erbel et al., 2006; Yildiz et al., 2013)
DENV3	3U1J, 3U1I	(Noble et al., 2012)
DENV4	2WZQ, 2VBC, 2WHX	(Luo et al., 2008; Luo et al., 2010)

However, ligands mostly originate from virtual libraries comprising thousands to millions of compounds. The most commonly used docking software in NS2B/NS3pro studies are AutoDock, AutoDock Vina, and Molecular Operating Environment (MOE). These platforms have an algorithm for identifying the NS2B/NS3pro active site by allowing small drug-like molecules to bind to different parts of the protein. The best ligand-protein affinity and binding positions were then observed (Jakhar et al., 2019). Nevertheless, it is essential to observe the hydrogen bonding and optimize the hydrophobic interactions, as they are the key players in obtaining stable energy-favored ligands at the interface of a protein structure and help in modifying the binding affinity for the drug’s effectiveness. Studies that have applied only *in silico*-based approaches to explore the interaction between NS2B/NS3pro and its possible inhibitor candidates in recent years are tabulated in **Table 3**.

**Table 3:** Summary of NS2B/NS3 protease inhibitors recent development applying *in silico* method.

	Compound name	Method	Docking score (kcal/mol)	Closed-contact residues	Ref.
**1**	Nimbin -Triterpenoids (From *Azadirachta* *indica* (neem))	i. Protein PDB ID: 2VBC ii.Ligands: Natural product compounds iii.Molecular docking program: MTiAutoDock	–5.56	His51, Asp75, Ser135, Asn152, Val36, Arg73, Pro132, Gly133, Gly153, Val154	(Dwivedi et al., 2016)
**2**	Desacetylnimbin -Triterpenoids (From *Azadirachta* *indica* (neem))		–5.24	Arg54, Gly133, Asn152, Val36, Trp50, His51, Val72, Arg73, Asp75, Pro132, Ser135	
**3**	Desacetylsalannin - Triterpenoids (From *Azadirachta* *indica* (neem))		–3.43	Trp50, His51, His54, Val72, Arg73, Asp75, Asn152	
**4**	ZINC ID: 75163069	i. Protein PDB ID: 2FOM ii. Ligands: Synthesized compounds from ZINC database iii. Pharmacophore modelling and Molecular docking program: Molecular Operating Environment (MOE)	-19.98	His51, Asp75, Ser135, Gly153, Gly151, Pro132, Val154, Leu128	(Qamar et al., 2016)
**5**	ZINC ID: 59170698		-18.26	His51, Asp75, Ser135, Lyc73, Gly153, Pro132, Arg54	
**6**	ZINC ID: 06395655		-20.08	His51, Asp75, Gly153, Gly151, Pro132, Tyr161	
**7**	ZINC ID: 32933073		-22.34	His51, Asp75, Ser135, Pro132, Gly153, Ile36	
**8**	ZINC ID: 13728171		-10.22	His51, Asp75, Tyr161, Gly153, Pro132, Ile36, Leu128, Gly151	
**9**	ZINC ID: 65395833		-19.89	His51, Asp75, Gly151, Leu128, Gly153	
**10**	Baicalein (flavonoid)	i. Protein PDB ID: 2FOM ii. Ligands: Synthesized compounds iii. Pharmacophore modeling and Molecular docking program: AutoDock Vina 1.5.6, Discovery Studio 2.5	-7.5	Lys74, Leu76, Asn152, Trp83, Leu149, Gly148, Glu88, Asn152, Leu149, Trp83	(Hassandarvish et al., 2016)
**11**	Baicalin (flavonoid)		-8.0	Gly148, Leu149, Trp83, Leu76, Asn152, Trp86, Leu128, Tyr161, Arg54, Gly153, Tyr161, His51, Tyr150	
**12**	Meclofenamic acid (Compound **4**)	i. Protein: 3D homology model of NS2B-NS3 protease of DENV-2, namely DH-1 retrieved from Heh et al. (2013). ii. Ligands: Synthesized compound from PubChem iii. Molecular docking program: AutoDock	-3.64	His51, Gly151, Val155, Tyr161, Phe130, Ser131, Pro132, Thr134, Ser135, Tyr150, Asn152, Gly153, Val154	(Othman et al., 2017)
**13**	Rolitetracycline (Compound **5**)		-3.21	Gly153, Phe130, Gly151, Tyr161, Asn152, His51, Asp129, Thr134, Ser135, Tyr150, Val154, Pro132, Val155	
**14**	Uncinanone B (Plant flavonoid; C_20_H_18_O_6_)	i. Protein PDB ID: 2FOM ii. Ligands: Natural product compounds from MAPS database Pubchem Zinc database, ChEBI, MPD3 and ChEMBL	-12.156	His51, Pro132, Asp75, Gly153, Leu128, Ser135	(Qamar et al., 2017)
**15**	5- hydroxybowdichione (Plant flavonoid; C_16_H1_0_O_7_)		-12.110	His51, Tyr150, Asp75, Gly153, Ser135, Pro132, Leu128	
**16**	Prunetin (Plant flavonoid; C_16_H_12_O_5_)		-11.369	His51, Tyr150, Asp75, Gly153, Leu128, Pro132	
**17**	5,7,3’,4’- tetrahydroxyisoflavone (Plant flavonoid; C_21_H_20_O_11_)		-10.534	His51, Pro132, Gly153, Leu128, Ser135, Asp75	
**18**	Alpinumisoflavone (Plant flavonoid; C_20_H_16_O_5_)		-10.449	His51, Gly153, Asp75, Pro132, Leu128	
**19**	Glicoisoflavanone (Plant flavonoid; C_20_H_18_O_6_)		-10.015	His51, Asp75, Pro132, Leu128, Gly153	
**20**	Fumaritine N-oxide (*Fumaria indica*)	i. Protein PDB ID: 2FOM ii. Ligands: Natural product compounds from PubChem iii. Molecular docking program: AutoDock Vina	-9.2	His51, Arg54, Val72, Asp75, Asn152	(Rasool et al., 2018)
**21**	Osajin (*Erythrina variegate*)		-9.7	Leu128, Phe130, Pro132, Tyr150, Gly151, Gly153	
**22**	SigmodinA		-9.0	His51, Leu128, Pro132	
**23**	SigmodinB		-9.4	His51, Asp75, Leu128, Pro132, Val154	
**24**	SigmodinC		-9.4	His51, Asp75, Leu128, Pro132, Ser135, Gly153	
**25**	SKYa 4-Thiazolidinone coumarin derivatives	i. Protein PDB ID: 2FOM ii. Ligands: Synthesized compounds iii. Molecular docking program: Receptor Grid Generation™	-2.754	His51, Asp75, Tyr150, Gly151, Asn152, Gly153, Ser135, Pro132, Ser131, Phe130, Leu128	(Yusufzai et al., 2018a)
**26**	SKYb 4-Thiazolidinone coumarin derivatives		-2.960	Asp75, Val154, Gly153, Asn152, Gly151, Tyr150, His51, Leu128, Phe130, Ser131, Pro132, Ser135	
**27**	SKYc 4-Thiazolidinone coumarin derivatives		-3.905	His51, Gly153, Gly151, Tyr150, Leu128, Phe130, Ser131, Pro132, Ser135	
**28**	Quercetin 3-O-(2′′,3′′-digalloyl)-β-D-galactopyranoside (*Euphorbia lunulata*)	i. Protein PDB ID: 2FOM ii. Ligands: Natural product compounds from Chebi database iii. Molecular docking program: Molecular Operating Environment (MOE)	-26.101	Gly87, Val146, Asn167	(Sarwar et al., 2018)
**29**	Quercetin 3-O-α- (6′′’-caffeoylglucosyl-β-1,2-rhamnoside) (*Sedum sarmentosum*)		-24.987	Lys74, Ile165	
**30**	Schaftoside (*Passiflora tripartita*)		-23.399	Trp83	
**31**	Myricetin (*Myrica rubra*)		-21.987	Trp83, Gly87, Val146	
**32**	Quercetin 3-sulfate (*Anethum graveolens*)		-20.989	Lys74	
**33**	Eriocitrin (*Citrus lumia, Cyclopia subternata*)		-20.693	Lys74	
**34**	Catiguanin B (*Trichilia catigua*)		-20.414	Lys74, Trp83	
**35**	4′,5,7-trihydroxy-3-methoxyflavone-7-O- α-L-arabinofuranosyl(1 → 6)-β-D-glucopyranoside (*Lepisorus contortus*)		-20.378	Asn67, Val47, Trp89	
**36**	Wogonin 7-O-β-D-glucuronide (*Scutellaria baicalensis*)		-20.102	Gly87, Trp83	
**37**	Silychristin (*Silybum marianum*)		-20.085	Lys74, Trp83	
**38**	(E)-7-Hydroxy-3-(1-(2-(4-p-tolylthiazol-2-yl)hydrazono) ethyl)-2H-chrom-en-2-one (Compound 7c)	i. Protein PDB ID: 2FOM ii. Ligands: Synthesized compounds iii. Molecular docking program: Receptor Grid Generation^TM^	-5.141	Ser131, Pro132, Ser135, Gly151, Gly153, Asp75, Val72, Trp50, His51, Tyr161, Leu128, Tyr150	(Yusufzai et al., 2018b)
**39**	(E)-7-Methoxy-3-(1-(2-(4-phenylthiazol-2-yl)hydrazono) ethyl)-2H-chromen-2-one (7l) (Compound 7l)		-3.894	Gly153, Asp75, Val154, Leu154, Leu128, Phe130, Tyr150, Ser131, Pro132, Ser135, Gly151, Val72, Lys73	
**40**	Indanone derivatives (Compound 3g)	i. Protein: Homologous crystal structure by Wichapong et al. (2010) ii: Ligand: Synthesized compounds iii. Molecular docking program: AutoDock 4.2	–7.3	Gly82, His51, Tyr161	(Nesfu et al., 2019)
**41**	Indanone derivatives (Compound 3h)		–7.3	Asn154, His51, Tyr161	
**42**	Ganodermanotriol (Triterpenoids of *Ganoderma lucidum*)	i. Protein PDB ID: 2FOM ii: Ligand: Natural product compounds iii. Molecular docking program: Schrodinger	−6.291	Lys73, Thr120, Asn167, Trp50, Val72, Ile123, Val154, Val155, Ala164, His51, Thr118, Asn119, Asn152, Gly153, Lys74, Asp75	(Bharadwaj et al., 2019)
**43**	Canthin-6-one 9-O-beta-glucopyranoside	i. Protein PDB ID: 2FOM ii: Ligand: Natural product compounds from MPD3 database, MAPS database, Pubchem and Zinc database iii. Molecular docking program: MOE	−15.17	His51, Asp75, Ser135, Gly151, Gly153, Asn152, Leu128, Tyr150	(ul Qamar et al., 2019)
**44**	Kushenol W		-14.55	His51, Ser135, Gly151, Gly153, Asp75, Tyr161, Leu128, Asn152, Pro132, Phe130	
**45**	Kushenol K		−16.39	His51, Ser135, Pro132, Tyr150, Asp75, Gly153, Leu128, Gly151, Asn152, Phe130	
**46**	(3E,5E)-3,5-bis(4-methoxybenzylidene)-1-(phenylsulfonyl) piperidin-4-one (Compound 2)	i. Protein PDB ID: 2FOM ii: Ligand: Synthesized compounds iii. Molecular docking program: AutuDock	-61.01	Gly51, Arg54, Pro132, His51, Asp75 and Ser135	(Ikhtiarudin, 2019)
**47**	Orientin (Phytoconstituents of Cynodon dactylon)	i. Protein PDB ID: 3U1I ii: Ligand: Natiral product compounds from PubChem iii. Molecular docking program: FlexX Lead IT 2.3.	-21.9439	Asn B:152, Tyr B:16, Gly B:151, Gly B:153, Phe B:130, Lys B:131, Arg B:54, His B:51	(Chandani et al., 2019)
**48**	Triglochinin (Phytoconstituents of *Cynodon dactylon*)		-29.0361	Gly B:153, Gly B:133, Gly B:151	
**49**	Apigenin (Phytoconstituents of *Cynodon dactylon*)		-26.2859	His B: 51, Phe B:130, Tyr B:150, Ser B:135	
**50**	Luteolin (Phytoconstituents of *Cynodon dactylon*)		-29.4214	Lys B:131, Tyr B:150, Gly B:153	
**51**	Fluorinated pyrazoline analogue (Compound 1)	i. Protein PDB ID: 2FOM ii: Ligand: Synthesized compound iii. Molecular docking program: MOE	−59.98	His51, Arg74 Asp75 His51, Arg	(Zamri et al., 2019)
**52**	Coumarin derivatives (Compound Vb)	i. Protein PDB ID: (ND) ii: Ligand: Synthesized compound iii. Molecular docking program: iGEMDOCK	-104.22	Gly151, Tyr150	(Tataringa et al., 2019)
**53**	Luteolin (Phytochemical of *Carica papaya*)	i. Protein PDB ID: 2FOM ii: Ligand: Natural product compound iii. Molecular docking program: PyRx software (Version 0.8)	-7.7	Asp75, Gly153, Ser131, Leu128, Phe130, Tyr150	(Ghosh and Talukdar, 2019)
**54**	Epigallocatchin (*Carica papaya* bioactive compound)	i. Protein PDB ID: (ND) ii: Ligand: Natural product compounds iii. Molecular docking program: MOE	−13.2911	His51, Asp75, Ser135, Val72, Lys73, Tyr135, Gly151	(Farooq et al., 2020)
**55**	Catechin (*Carica papaya* bioactive compound)		−9.0122	His51, Asp75, Ser135, Val72, Lys73, Tyr135, Gly151	
**56**	Protocatechuric acid (*Carica papaya* bioactive compound)		-7.5592	His51, Asp75, Ser135, Val72, Lys73, Tyr135, Gly151	
**57**	C_25_H_21_N_5_O_3_ (Compound A1)	i. Protein PDB ID: 2FOM ii: Ligand: Synthesized compounds from Asinex database iii. Molecular docking program: AutoDock Vina	-10.86	Thr120, Asn152, Asn167, Val72, Leu76, Ile123, Leu76	(Bhowmick et al., 2020)
**58**	C_21_H_19_FN_6_O_2_ (Compound A2)		-11.07	Asn167, Asn152, Lys73, Leu76, Al164	
**59**	C_23_H_20_N_6_O_2_ (Compound A3)		-10.97	Lys73, Asn152, Asn167, Lys74, Leu76, Ile123, Ala164	
**60**	C_21_H_19_FN_4_O_4_ (Compound A4)		-10.71	Lys73, Gly153, Asn167 Lys74, Leu76	
**61**	C_28_H_35_N_5_O_4_ (Compound A5)		-10.33	Thr120, Asn152, Gly153	
**62**	CAA15	i. Protein PDB ID: Model - homologous crystal structure of DENV-2 NS2B/NS3pro ii: Ligand: Synthesized compounds from Asinex database iii. Molecular docking program: AutoDock 4.2	-7.22	Ile36, His51, Al52, Asp29, Phe130, Pro132, Tyr161	(Puc et al., 2021)
**63**	CAA16		-7.03	Ile36, His51, Val52, Asp29, Phe130, Pro132, Tyr161	
**64**	CAA17		-7.07	Val52, Arg54, Asp29, Phe130, Pro132, Tyr161	

In conclusion, this approach determines the best-fitting ligand positions in the NS2B/NS3pro binding pocket and estimates the affinity of the ligand to the protein. The *in silico* approach uses crystal structures of the DENV NS2B/NS3pro protein with various PDB codes as well as ligands from virtual libraries containing hundreds to millions of chemicals. The ideal ligand-protein affinity and binding location can be determined using the software. However, the crucial point is that many chemical compounds and peptides have shown significant *in silico* binding affinity towards viral targets, but their affinity has yet to be evaluated using *in vitro* methods in many cases. Hence, the mechanism underlying the inhibition of most peptides remains unknown.

## 4 *In vitro* approach

**Figure 4:**
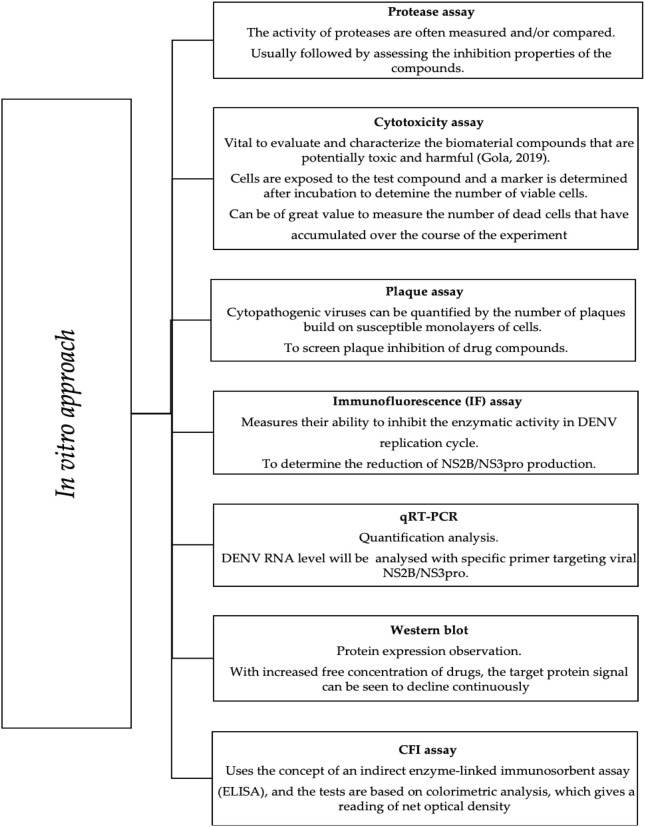
*In vitro* approach in DENV NS2B/NS3 pro inhibition study.

According to Lim et al., virtual hits derived from *in silico* docking require further validation by *in vitro* methods. These methods can verify on-target effects in cells (Lim, 2019). The *in vitro* assays are commonly performed to investigate the inhibitory properties of candidates against NS2B/NS3pro, as briefly described in **Figure 4**. Plaque, cytotoxicity, and immunofluorescence (IF) assays are examples of cell-based assays that provide substantial information on various cellular responses to compound exposure. Therefore, choosing the right cell type based on the target biology is critical. Among the cell types used in recent dengue inhibition studies, Vero, E6, C6/36, and BHK21 cells are effective for DENV propagation (Phanthanawiboon et al., 2014).

According to the Guidance for Industry-Antiviral Product Development by the US Food and Drug Administration (FDA), the specific antiviral activity was quantitatively measured by calculating the replication of the virus in the presence of increasing drug concentrations as opposed to replication in the absence of the drug. Therefore, to evaluate drug potency, the inhibition concentration (IC_50_) and effective concentration (EC_50_) must be measured (FDA, 2006). Nearly all recent studies have reported the IC_50_ of the tested compounds and their activities against the dengue enzyme. Quantification was performed using a protease inhibition assay, which measures the inhibitory activity of the drug candidates and the catalytic activity of the proteolytic enzyme.

As mentioned previously, *in vitro* drug potency measurements are essential for drug discovery. We reviewed recent studies that evaluated the EC_50_ of inhibitors through cytotoxicity tests (Chu Lee et al., 2015; Li et al., 2015; Lim et al., 2020) or plaque assays, such as the time of drug addition (Li et al., 2018; Yao et al., 2018), viral plaque reduction (Brecher et al., 2017; Li et al., 2018), and viral titer reduction assay (Brecher et al., 2017).

In addition to identifying an effective drug, it is crucial to determine the cytotoxic potential of the tested compounds in the drug discovery process (Slater, 2001). Briefly, the cytotoxic concentration (CC_50_) of the compounds that caused a reduction in cell viability was measured using a dilution assay. In recent studies, MTT (Raut et al., 2015b; Cabarcas-Montalvo et al., 2016; Beesetti et al., 2018; Li et al., 2018) or other cytotoxicity assays have been used to observe their effects on virus-infected host cells. The antiviral activity of the compounds was tested at different concentrations. Hence, developing potential inhibitors with lower cytotoxic concentrations is recommended (Alagarasu et al., 2022). In conclusion, many DENV NS2B/NS3 pro-inhibitor candidates have yet to be subjected to cytotoxicity investigation, making these products uncertain for further development.

Protease assays are another key pre-clinical assay in investigating protease inhibitors and their activity. For most inhibitor candidates, the target enzymatic activity was quantitatively determined to test their efficacy against the NS2B/NS3 pro-enzyme (Raut et al., 2015b; Brecher et al., 2017; Osman Idris et al., 2017; Beesetti et al., 2018; Euanorasetr et al., 2019; Hariono et al., 2019; Lim et al., 2020; Saleem et al., 2019; Sulaiman et al., 2019). The activity was determined if the tested compounds modulated the DENV NS2B/NS3 pro-enzyme function. Here, we highlight the recent five-year studies that applied only *the in vitro* approach (**Table 4**) and a combination of *in vitro* and *in silico* approaches (**Table 5**) to determine protease enzymatic activity.

**Table 4:** Summary of NS2B/NS3 protease inhibitors recent development applying *in vitro* method.

	Compound	Method	Cells type	EC_50_(µM)	CC_50_ (µM)	IC_50_	Ref.
**1**	Curcumin derivative (CC3)	i. Compound synthesis ii. *In vitro* protease assay iii. BHK/DENV2 replicon assay iv.Cell-based cytotoxicity assay v.Plaque assays vi.qRT-PCR vii.Oil Red O staining of lipid droplets viii.Actin staining with phalloidi	BHK-21, LLC-MK2	2.68 ± 0.64	32.34 ± 4.72	39.17 ± 6.69 µM/ml	(Balasubramanian et al., 2019)
**2**	Curcumin derivative (CC4)		BHK-21, LLC-MK2	5.37 ± 0.62	87.40 ± 9.03	43.88 ± 10.14 µM/ml	
**3**	Curcumin derivative (CC5)		BHK-21, LLC-MK2	2.34 ± 0.21	25.50 ± 2.64	60.98 ± 8.7 µM/ml	
**4**	*Dryobalanops aromatic leaves* (methanol extract)	i. Extraction ii. Protease inhibition assay	ND	ND	ND	0.30 ± 0.16 μg/mL	(Salleh et al., 2019)
**5**	Spirotetronate compounds (2EPS-A) isolated from *Actinomadura* strain	i. Protease assay ii. Cytotoxocity test iii. Plaque assay iv.Virus quantification by plaque formation assay.	Vero	ND	ND	1.94 ± 0.18 μg/mL	(Euanorasetr et al., 2019)
**6**	Spirotetronate compounds (2EPS-B) isolated from *Actinomadura* strain		Vero	ND	ND	1.47 ± 0.15 μg/mL	
**7**	Spirotetronate compounds (2EPS-C) isolated from *Actinomadura* strain		Vero	ND	ND	2.51 ± 0.21 μg/mL	
**8**	Diaryl (thio)ethers derivatives (Compound 1)	i. Compound synthesis ii. Molecular docking iii. Fluorometric DENV protease assays iv. qRT-PCR v. Cell culture-based protease assay vi. Cell toxicity test	Vero	3.5 ± 0.3	15.6 ± 3.4	98 ± 4 μM	(Wu et al., 2015)
**9**	Diaryl (thio)ethers derivatives (Compound 2)		Vero	ND	ND	34 ± 5 μM	
**10**	Diaryl (thio)ethers derivatives (Compound 3)		Vero	0.1 ± 0.0	0.2 ± 0.0	22 ± 1 μM	
**11**	Diaryl (thio)ethers derivatives (Compound 4)		Vero	0.3 ± 0.1	0.7 ± 0.1	26 ± 1 μM	
**12**	Diaryl (thio)ethers derivatives (Compound 5)		Vero	0.9 ± 0.1	2.3 ± 0.7	66 ± 3 μ	
**13**	Diaryl (thio)ethers derivatives (Compound 6)		Vero	0.8 ± 0.2	3.2 ± 1.2	4.2 ± 0.44 μM	
**14**	Diaryl (thio)ethers derivatives (Compound 7)		Vero	2.5 ± 0.1	9.3 ± 2.5	10% inhibition at 50 μM	
**15**	Diaryl (thio)ethers derivatives (Compound 8)		Vero	>3	>3	3.6 ± 0.11 μM	

ND, Not defined.

**Table 5:** Summary of NS2B/NS3 protease inhibitors recent development applying *in vitro* and *in silico* method.

	Compound	*In vitro* method					*In silico* method			
		Method	Cell type	EC_50_ (µM)	CC_50_ (µM)	IC_50_ (µM)	Method	Docking energy (kcal/mol)	Residues interacting with Ligand	Ref
1	MB21	i. Protease inhibition assays ii. Cell-based DENV inhibition assay iii. MTT assay iv. Molecular docking	Vero	ND	ND	5.95 μ	i. Protein PDB ID: 2FOM ii. Ligands: Synthesized compound from ‘In-house’ library iii. Molecular docking program: Glide v5.7	ND	Ile123, Val147, Tro83, Leu85, Ile165, Leu76, Met46, Ala164, Val154, Val155	(Raut et al., 2015a)
2	T5341917 (Compound 14)	i) Molecular docking ii) Protease inhibition assay iii) Cell-based flavivirus immune detection iv) Cell viability assay	Huh-7 and BHK21	5.0 ± 0.2 (HuH7), 5.0 ± 1.1 (BHK21)	>300 (HuH7), 55.0 (BHK21)	85% mean inhibition	i. Protein PDB ID: 3U1I ii. Ligands: Synthesized compounds from ChemBridge library iii. Molecular docking program: MOE, AutoDock	-10.65	Pro132, Val155, Tyr161, Met84, Gly153, Ile86, Val165	(Li et al., 2015)
3	C_35_H_27_NO_9_ (CID 54681617)	i) Molecular docking ii) Fluorimetric enzyme activity assay iii) MTT assay iv) Virus yield reduction assay	HepG-2	ND	58.6 ± 3.0	14.9 ± 2.9	i. Protein PDB ID: 2FOM ii. Ligands: Synthesized compounds from PubChem iii. Molecular docking program: AutoDock Vina	-11.6	Ile65, Trp69, Lys74, Leu76, Thr120, Ile123, Val154, Ala164, Ile165, and Ala166	(Cabarcas-Montalvo et al., 2016)
4	C_30_H_25_NO_5_ (CID 54692801)	i) Molecular docking ii) Fluorimetric enzyme activity assay iii) MTT assay iv) Virus yield reduction assay		ND	42.1 ± 1.6	11.8 ± 0.2		-13.5	Ile65, Trp69, Lys74, Leu76, Thr120, Ile123, Val154, Ala164, Ile165, and Ala166	
5	C_34_H2_3_NO_7_S_2_ (CID 54715399)	i) Molecular docking ii) Fluorimetric enzyme activity assay iii) MTT assay iv) Virus yield reduction assay		ND	162.4 ± 0.9	61.5 ± 4.6		-11.4	Ile65, Trp69, Lys74, Leu76, Thr120, Ile123, Val154, Ala164, Ile165, and Ala166	
6	Nitro derivatives of 3,5-bis(arylidene)-4-piperidones (Compound 4e)	i) Compound synthesis ii) Molecular docking iii) Protease assay	ND	ND	ND	15.22	i. Protein PDB ID: 2FOM ii. Ligands: Synthesized compounds iii. Molecular docking program: AutoDock	11.36	His51, Pro132, Ser135, Gly153 and Arg54	(Osman Idris et al., 2017)
7	Nitro derivatives of 3,5-bis(arylidene)-4-piperidones (Compound 4j)		ND	ND	ND	16.23		11.09	His51, Pro132, Ser135, Gly153, Arg54, Trp50	
8	NSC135618	I) Protease inhibition assay ii) Cytotoxicity assay iii) Viral titer reduction assay iv)Immunofluorescence assay v) qRT-PCR vi)Protein thermal shift assay vii) Western blot viii)Mass spectrometry	A549	0.81	48.8	1.8	i. Protein PDB ID: 2FOM ii. Ligands: Synthesized compounds from Diversity Set II library from the National Cancer Institute Developmental Therapeutics Program (NCI DTP) iii. Molecular docking program: AutoDock Vina	ND	Lys74, Asn152, Trp89, V147, Ala164, Val154, Ile123, Asn167, Trp89, Ile165, Ile147, Trp83, Leu149 and Leu76	(Brecher et al., 2017)
9	Calmodulin antagonist: N-(6-aminohexyl) - 5- chloro-1-naphthalene-sulfonamide hydrochloride (W-7)	i) Cell-based assay ii)Western blot iii) Confocal microscopy and flow cytometry (FACS) assays iv)qRT-PCR v)Molecular docking	Huh-7	ND	(W7 is not inducing apoptosis in Huh-7 cells)	(64% secretion reduction of NS3)	i. Protein PDB ID: ND ii. Ligands: Synthesized compound iii. Molecular docking program: Molegro Virtual Docker	92.502	His51, Asp75, and Ser135	(Bautista-Carbajal et al., 2017)
10	6-fluoro-4-(2-((5-nitrobenzo[d]thiazol-2-yl) amino)-2-oxoethoxy) quinoline-2- carboxylic acid (BT24)	i) Protease inhibition assay ii) Cell-based DENV inhibition assay iii) RT-PCR iii) plaque assay iv) MTT assay v) Molecular docking	Vero	ND	75.00	0.50	i. Protein PDB ID: 2FOM ii. Ligands: Synthesized compound from ‘in-house’ library, iii. Molecular docking program: Glide v5.7	ND	Trp83, Thr120 and Asn152	(Beesetti et al., 2018)
11	Diasarone-I	i. Virus-induced cytopathic effect and measurement of viral infection ii.Plaque assay iii. Time of drug addition assay iv. NS2B/NS3 enzyme inhibition assay v. Reactive oxygen species assay vi. Western blotting vii. Immunofluorescence assay viii. Quantitative real-time PCR (qRT-PCR) ix. Molecular docking	C6/36	4.5	>80	ND	i. Protein PDB ID: ND ii. Ligands: Natural product compounds iii. Molecular docking program: AutoDock Vina	-7.200	Lys105, Thr104, Gly83, Cys82, Gly81, Val132, Phe133, Ile141	(Yao et al., 2018)
12	N-(adamantan-1-yl)-4-[(adamantan-1-yl) sulfamoyl] benzamide) (Compound 3)	i. Compound synthesis ii. Cell-Based Flavivirus Immunodetection (CFI) Assay iii. Cytotoxicity Assay iv. Molecular docking	A549	ND	<100	22.4 ± 7.7	i. Protein PDB ID: 2FOM ii. Ligands: Synthesized compounds iii. Molecular docking program: MOE	-7.413	His51, Gly153	(Joubert Foxen and Malan, 2018)
13	N-(adamantan-1-yl)-4-sulfamoyl benzamide (Compound 7)		A549	ND	<100	42.8 ± 8.6		-7.123	Val72, Asp75, Gly153	
14	Erythrosin B	i. Protease inhibition assay ii. MTT assay iii. Viral reduction assay iv. IF assay v. qRT-PCR vi. Western blot vii. Molecular docking viii. Protein thermal shift assay (PTSA)	A549	1.2 ± 0.2	> 150	15	i. Protein PDB ID: 3U1I ii. Ligands: Synthesized compound iii. Molecular docking program: Schrodinger	ND	ND	(Li et al., 2018)
15	Thiosemicarbazones derived phenyl-acetyl ketones (DB-TYR-TSC)	i. Cytotoxicity assay ii. Indirect immunofluorescence assay iii. *In silico* method iv. Plaque formation unit reduction assay v. Molecular docking	Vero	ND	350	50	i. Protein PDB ID: 3U1I ii. Ligands: Synthesized compound iii. Molecular docking program: AutoDock 4.2.6 and Rasmol	-6.36	Ser135, Gly151, Pro132, Asp 75	(Padmapriya et al., 2018)
16	Thioguanine derivatives (Compound 18)	1. Compound synthesis 2. Molecular docking 3. Protease Inhibition assay Molecular dynamic simulation	ND	ND	ND	0.38	i. Protein PDB ID: 2FOM ii. Ligands: Synthesized compound from National Cancer Institute database, Hyperchem 8.0 iii. Molecular docking program: AutoDock4.2	-16.10± 2.70	Gly175, Asn174, Tyr183, Asp97, Tyr183, Ser157, Gly35, Ser36, His73, Asp34, Met37, Arg76	(Hariono et al., 2019)
17	Thioguanine derivatives (Compound 21)		ND	ND	ND	16		-18.24 ± 4.66	His73, Ser157, Asp97, Gly175, Asn174	
18	4-hydroxy-6-(9,13,17-trimethyldodeca- 8,12,16-trienyl)2(3H)-benzofuranone (Compound 1) (Isolated from *Endiandra kingiana*)	i) Protease activity assay ii) Molecular docking	ND	ND	ND	403.14 ± 33.03	i. Protein PDB ID: 2FOM ii. Ligands: Natural product compounds iii. Molecular docking program: AutoDock	ND	Asp129 and Ser135	(Sulaiman et al., 2019)
19	(−)-Epicatechin (Compound 2) (Isolated from *Endiandra kingiana*)		ND	ND	ND	170.10 ± 5.94		ND	Asp129, Ser135, Tyr161 and Asn152	
20	(+)-Catechin (Compound 3) (Isolated from *Endiandra kingiana*)		ND	ND	ND	184.13 ± 2.11		ND	Asp129, Tyr161 and Asn 152	
21	Hesperetin (From *Ganoderma lucidum* var. antler)	i) Protease activity assay ii) Cytotoxicity test iii) Molecular docking	WRL-68	326.	ND	ND	i. Protein PDB ID: 2FOM ii. Ligands: Natural product compounds from numerous molecular databases (ZINC, PubChem etc), GaussView 5.0 iii. Molecular docking program: HADDOCK2	- 7.2	His107, Val128, Pro188, Ser191, Trp106, Gly207, Asn208, Gly209, Tyr217, His107, Val128, Asp131, Leu184, Pro18 8, Gly207, Gly209, Tyr217 and Asp131	(Lim et al., 2020)
22	Isobiflorin (Compound 1) (From *S. aromaticum*) (cloves extract)	i) Protease activity assay ii)Protease inhibition assay iii) Molecular docking	ND	ND	ND	58.9 ± 1.3	i. Protein PDB ID: 3U1I ii. Ligands: Natural product compounds iii. Molecular docking program: AutoDock	−6.8	Trp-50, Arg-54, Asp-75, His-51, Val-72, Asp-81, and Asn-152	(Saleem et al., 2019)
23	Biflorin (Compound 2) (From *S. aromaticum*) (cloves extract)		ND	ND	ND	89.6 ± 4.4 μM		−7.2	Met-84, Ile-86, Asn-152, Gly-153, Tyr-161, Thr-83, Arg-85, Val-154, and Val-155	
24	Eugeniin (Compound 3) (From *S. aromaticum*) (cloves extract)		ND	ND	ND	94.7 ± 2.5 μM		−10.2	Asp-75, Asp-81, Met-84, Asp-129, Phe-130, Gly-133, Ser-135, His-51, Arg-54, Pro-132, Tyr-150, Val-154, Val-155, and Tyr-161	
25	Kaempferol-3-O-rutinoside (bioflavonoids from *Azadirachta indica*)	i) Molecular docking ii) Cytotoxicity test iii)Protease inhibition assay iv) IF assay	BHK-21	ND	No significant cyto-toxicity till 100 μM concentra-tion	55.6% in DENV-2 infectivity at lower concentra-tions of 1 and 10μM; Maximum inhibition of 77.7% at 10 and 100 μM concentra-tion	i. Protein PDB ID: 2FOM ii. Ligands: Natural product compounds iii. Molecular docking program: GLIDE5.8	–9.555	Asp75, Phe130, Gly151, Asn152, Gln153, Trp50, His51, Val72, Lys73, Leu128, Ser131, Pro132, Ser135, Tyr150, Val154, and Try161	(Dhar Dwivedi et al., 2020)
26	Epicatechin (Bioflavonoids from *Azadirachta indica*)		BHK-21	ND	20% cyto-toxicity on the BHK-21 cells at 1 mM (1000 μM) concentra-tion	47.1% reduction in the DENV-2 infectivity at 0.1 mM (100 μM); Maximum of 66.2% inhibition of DENV-2 infectivity at 1 mM (1000 μM) concentra-tion		-7.622	His51, Pro132, Gly151, Phe130, Leu128, Ser131, Gly133, Ser135, Try150, Asn152, Gly123, His51, Ser131, Ser135, Asn152, Gly133, Gly151, and Gly 153	
27	C_26_H_19_F_3_N_4_O_5_S_2_ Compound 8g	i) Compound synthesis ii) Protease activity assay iii) Protease inhibition assay iv) Molecular dockin	ND	ND	ND	13.9 ± 1.4	i. Protein PDB ID: 3U1I ii. Ligands: Synthesized compounds iii. Molecular docking program: AutoDock Vina	−8.8	Thr118, leu85, Trp83, Asn167	[106]
28	C_27_H_21_F_3_N_4_O_5_S_2_ Compound 8h		ND	ND	ND	15.1 ± 1.3		−8.8	Trp83, Asn167	
29	Compound 1	i. Molecular docking ii. Protease inhibition assay iii. Cell viability assay iv. Western blot, RT-PCR v. IF microscopy	Huh-7	ND	35.4mM	7.1mM	i. Protein PDB ID: 5YW1 ii. Ligands: Maestro v.11.5 iii. Molecular docking program: Schrodinger Suite v.2018	ND	Pro132, Tyr150, Tyr161, Asp129, Asp75.	(Shin et al., 2021)

ND, Not defined.

By using the *in vitro* methods, the on-target effects in cells can be verified using *in vitro* methods. The candidate inhibitory activities against NS2B/NS3pro were evaluated using a protease inhibition assay. Moreover, plaque, cytotoxicity, and IF assays can provide valuable information on the diverse cellular responses to compound exposure. The pharmacological potency of drugs can be assessed by quantitatively measuring their specific antiviral activity. However, the cytotoxicity assessment of inhibitors during drug potency evaluation is limited, leading to uncertainty in the further development of drugs.

## 5 Using a combination of methods

In summary, incorporating *in silico* and *in vitro* approaches to determine the potency of dengue inhibitors can lead to the development of more potential drug candidates. Furthermore, integrating *in vitro* methods with *in vivo* assessments will reduce the number of physiologically relevant potential candidates and evaluate their characteristics simultaneously. It will also evaluate drug-drug interactions (DDI) and help comprehend the underlying mechanisms of drug candidates. Additionally, combining these approaches will help verify the relevance of *in vitro* results. Thus, substantiating the extrapolation of *in vitro* outcomes to the clinical phase of the drug development pipeline.

In addition, we would like to highlight the sources of DENV NS2B/NS3 pro candidates. In our study, the small-molecule or non-peptide candidates explored were either synthetic (25 studies) or derived from natural sources (16 studies). Currently, most medicines used in clinical practice are synthetically formulated and include chemical processes (reactions) and phytochemicals. The four anti-DENV drugs under clinical trials, celgosivir, UV4B, chloroquine, and balapiravir (Anasir et al., 2020), are small synthetic molecules developed from natural sources. As synthetic drugs have benefits such as chemical purity, a simple and cost-effective preparation process, and higher quality, more effective and safer drugs can be prepared by altering the chemical structure of the drug prototype.

Alternatively, using natural sources is a well-established method for discovering new substances with possible therapeutic effects. This class of drugs comprises new bioactive compounds that are essential for the production of modern medicines (Kumar et al., 2019). It also provides information on different classes of bioactive lead compounds for the discovery and development of novel drugs. Recent studies have focused on the bioactive compounds present in plants, such as *Carica papaya* (Ghosh and Talukdar, 2019; Farooq et al., 2020), *Azadirachta indica* (Dwivedi et al., 2016), *Ganoderma lucidum* (Bharadwaj et al., 2019), *Ganoderma lucidum* var. antler (Lim et al., 2020), *Curcuma longa* (Balasubramanian et al., 2019), *Endiandra kingiana* (Sulaiman et al., 2019), *Cynodon dactylon* (Chandani et al., 2019), *Dryobalanops aromatic*um (Salleh et al., 2019), *Acorus tatarinowii Schott* (Yao et al., 2018), and *Syzygium aromaticum* (Saleem et al., 2019). The above mentioned studies included the extraction of crude plants (or plant parts) in solvents, mainly methanol, before investigating its activity against DENV NS2B/NS3 pro. Nonetheless, from our observation, both cohorts led to potent inhibitors with promising activity against the DENV NS2B/NS3 proenzyme, which has the potential to progress to the next anti-DENV drug development phase.

## 6 Conclusion

The number of hits, particularly those obtained from *in silico* docking, should be verified using *an in vitro* approach. It is also important to fully characterize the hits identified from the compound libraries. Furthermore, to produce a promising DENV antiviral inhibitor, verifying its activity using *in vitro* methods is crucial. To further ascertain the outcome of the two approaches, incorporating *in vivo* assessments can be beneficial, as they can substantiate the *in vitro* outcomes to the clinical phase in the drug development pipeline. These combined approaches can lead to promising antiviral candidates that may curb dengue infection. Additionally, along with small drug-like molecules, the search for dengue inhibitors should focus on using peptides. As signaling molecules, this possible approach exhibits complex biological roles with high selectivity and comparatively safe criteria.

Furthermore, we emphasize that a DENV inhibitor must be effective against all four DENV serotypes, as these serotypes co-circulate in highly endemic regions [95]. Nevertheless, it is essential to remember that the plausibility of dengue serotypes, together with other factors, such as secondary infection by a heterologous serotype, age, comorbidity, poor clinical prognosis, diagnosis, virulence, and the host immune response, contribute to the development of severe dengue infection (Puc et al., 2021). Finally, considering the recent attempts to identify DENV NS2B/NS3pro inhibitors, a range of antiviral targets display antiviral intervention potential. Although small-molecule inhibitors require clinical approval, promising dengue antivirals will be possible soon.

## Author contributions

LS designed the study, HN carried out the data collection, HN, KE, LH, data analysis and interpretation. LS. and HN, drafted the article. HN, RV, RA and AH edited the article. All authors read and approved the final article. Authors contributed equally for the preparation of this review.
